# Development of a Liquid Chromatography–High Resolution Mass Spectrometry Method for the Determination of Free Fatty Acids in Milk

**DOI:** 10.3390/molecules25071548

**Published:** 2020-03-28

**Authors:** Maroula G. Kokotou, Christiana Mantzourani, George Kokotos

**Affiliations:** Department of Chemistry, National and Kapodistrian University of Athens, Athens 15771, Greece; mkokotou@chem.uoa.gr (M.G.K.); chrmantz@chem.uoa.gr (C.M.)

**Keywords:** determination, free fatty acids, high resolution mass spectrometry, liquid chromatography, milk

## Abstract

The determination of free fatty acids (FFAs) in milk is of importance for quality control, legislative purposes, authentication and product development. We present herein a liquid chromatography–high resolution mass spectrometry method for the direct determination of FFAs in milk. The method involves mild sample preparation, avoids time-consuming derivatization and allows the direct quantification of twenty-two FFAs in a 10-min single run. It was validated and applied in thirteen cow milk and seven goat milk samples. Saturated fatty acids C16:0, C18:0 and unsaturated C18:1 (n-9) were found to be the major components of milk FFAs at concentrations of 33.1 ± 8.2 μg/mL, 16.5 ± 5.3 μg/mL and 14.8 ± 3.8 μg/mL, respectively, in cow milk and at concentrations of 22.8 ± 1.8 μg/mL, 12.7 ± 2.8 μg/mL and 13.3 ± 0.3 μg/mL, respectively, in goat milk. Other saturated and unsaturated FFAs were found in significantly lower quantities. Saturated fatty acids C6:0, C8:0 and C10:0 were found in higher quantities in goat milk than in cow milk. The levels of the important (for human health) odd-chain FFAs C15:0 and C17:0 were estimated in cow and goat milk.

## 1. Introduction

Triacylglycerols (TAGs) are the dominant component of milk fat (accounting for >95% of the total lipid content), accompanied by small amounts of di- and mono-acylglycerols, free fatty acids (FFAs), phospholipids and cholesterol [1,2]. The presence of FFAs in milk is of great importance, because FFAs have strong sensory properties contributing to the flavor and aroma of milk. FFAs are primarily formed in milk and other dairy products through the breakdown of TAGs, due to the enzymatic hydrolysis by lipoprotein lipase and other lipolytic enzymes [3,4,5]. Elevated levels of FFAs are responsible for rancidity in milk and FFAs levels exceeding 1.5 mmol/L are generally unacceptable for the consumer [5]. Furthermore, some of the beneficial health and nutritional effects of milk may be attributed to particular FAs [6]. Therefore, the determination of FFAs in milk is of importance for quality control, legislative purposes, authentication and product development.

The most common approach to quantify FFAs in dairy products is the use of gas chromatography flame ionization detection (GC-FID), involving the conversion of fatty acids into the corresponding methyl esters (FAME) [7,8]. A recent report compares three acid- or alkaline-catalyzed transesterification methods and proposes a simple one-step protocol based on 0.2 M methanolic KOH, a short reaction time (20 min) and a mild reaction temperature (50 °C) for milk FAME preparation [9]. The quantification of FFAs in milk has also been reported using an in-solution derivatization to ethyl esters by GC/MS [10]. Most recently, a butyl ester method was reported for the determination of FFAs in dairy products, where extracted free fatty acids were converted to butyl esters prior to GC-FID [11]. To our knowledge, liquid chromatography–mass spectrometry (LC/MS) methods on the determination of FFAs in milk that avoid a derivatization step do not exist, while LC/MS studies applied after derivatization are also very limited. La Nasa et al. have described the determination of FFAs in food samples, including milk, after derivatization with 2-hydrazinoquinoline, using HPLC-ESI-Q-ToF [12]. The determination of FFAs in infant milk powder employing a 2,4-dimethoxy-6-piperazin-1-yl pyrimidine (DMPP)-based isotope derivatization technique and UHPLC–ESI-MS/MS has been reported [13]. The direct determination of ten FFAs in cheese by applying matrix solid-phase dispersion followed by UPLC and tandem MS analysis has been described [14]. 

For decades, guidelines have suggested the reduction of dietary saturated FAs to lower the risk of metabolic syndrome and cardiovascular disease (CVD). In recent years, growing evidence from scientific studies has modified this notion for milk, leading to disestablishment of these recommendations in a healthy population [6]. The 2018 World Health Organization (WHO) draft guidelines on dietary saturated FAs recommend reducing total intake of saturated fat and replacing it with polyunsaturated and monounsaturated FAs [15]. However, recently Astrup and colleagues argued that these guidelines fail to take into account considerable evidence that the health effects vary for different types of saturated FAs and that the composition of the food source is crucially important [16]. It has been demonstrated that the high circulating odd-chain saturated fatty acid C17:0 is inversely associated with CVD and stroke mortality and potentially associated with higher risk of non-CVD death [17]. A recent large meta-analysis, which pooled the findings from 16 prospective cohort studies, has shown that higher levels of odd-chain saturated fatty acids C15:0 and C17:0 are associated with a lower risk of type 2 diabetes (T2D) [18].

The aim of our work was the development of a straightforward method for the rapid determination of a big set of FFAs in milk, permitting the estimation of the content of each particular FA. Herein, we describe a liquid chromatography–high resolution mass spectrometry (LC-HRMS) method for the direct determination of FFAs, avoiding any derivatization step and following a simple and mild liquid/liquid extraction protocol for sample preparation. The present method allows the simultaneous determination of twenty-two FFAs in a 10-min single run.

## 2. Results and Discussion

### 2.1. ESI-MS and LC Data

Twenty-two FAs, including short, medium and long chain saturated fatty acids, as well as monounsaturated and polyunsaturated fatty acids, were studied. The high-resolution mass spectra of these FAs were recorded in ESI negative mode. The structures of the standard compounds along with the exact masses of the deprotonated molecules, theoretical and measured (single measurement), and the mass errors are summarized in Table 1.

**Figure 1:**
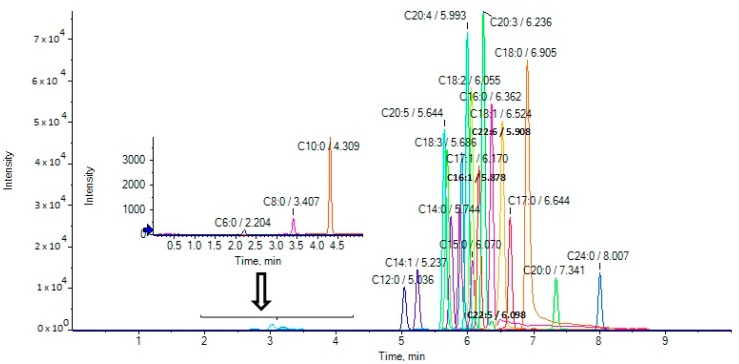
Extracted ion chromatograms (EICs) of fatty acids in a standard solution (500 ng/mL).

The extracted ion chromatograms (EICs) of the analytes in a standard solution (500 ng/mL) are presented in Figure 1. The retention times of the various analytes are included in Table 3.

### 2.2. Method Validation

The method was comprehensively validated to establish good linearity values for the analytes (R^2^ > 0.99), limits of detection (0.4–1.6 ng/mL) and limits of quantification (1.1–4.8 ng/mL). The calibration curve data as well as limits of detection (LOD) and quantification (LOQ) are presented in Table 2.

A simple liquid/liquid extraction protocol was used involving the addition of methanol for the protein precipitation. The EU Commission decision 202/657/EC was followed as a guideline for the verification of the accuracy and precision. Milk samples were spiked at three different concentration levels with three replicates for each fortification level. The proposed method was found to be accurate with satisfactory recoveries ranging from 82% to 97% for the low spike level and from 91% to 102% for the high spike level (Table 3). The precision was investigated by means of the relative standard deviation (%RSD). The %RSD values that were obtained for intra-day (RSDr) and inter-day (RSD_R_) variations ranged from 0.31 to 18.51 and from 1.15 to 19.12, respectively depending on the FA (Table 3).

### 2.3. Analysis of Samples

**Figure 2:**
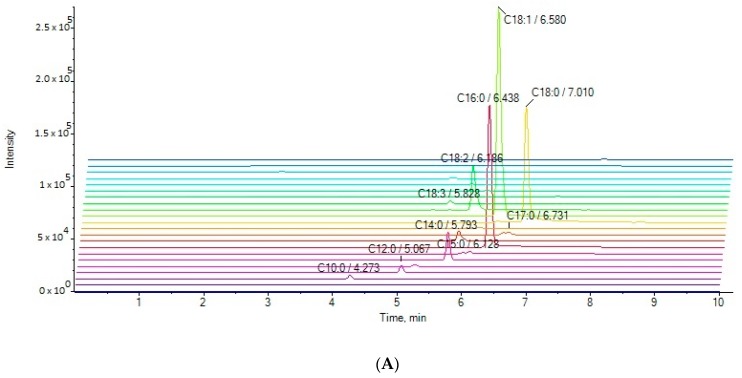

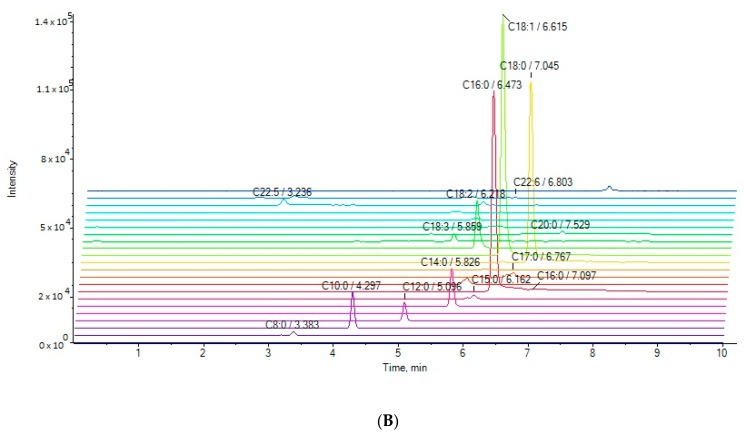
EICs of the analytes in a cow milk sample (**A**) and in a goat milk sample (**B**).

Thirteen cow milk samples and seven goat milk samples, which were purchased from the local market, were analyzed. The extracted ion chromatograms (EICs) of a cow milk sample (A) and a goat milk (B) are presented in Figure 2. The present method allows the simultaneous determination of twenty-two fatty acids in a 10-min single run. The contents of the twenty-two analytes in milk samples (in triplicate) are summarized in Table 4. The contents of FFA are expressed as μg of fatty acid per mL of milk.

Τhe long chain saturated FAs C16:0 and C18:0, as well as monounsaturated C18:1 (n-9), were found to be the most abundant FFAs in both cow and goat milk samples. The contents of the fatty acids C16:0, C18:0 and C18:1 (n-9) were estimated at mean values of 33.1 ± 8.2 μg/mL, 16.5 ± 5.3 μg/mL and 14.8 ± 3.8 μg/mL, respectively, in cow milk, while the corresponding values in goat milk were 22.8 ± 1.8 μg/mL, 12.7± 2.8 μg/mL and 13.3 ± 0.3 μg/mL, respectively. Following in abundance, the saturated FAs C12:0 and C14:0 were found at lower concentrations (3.9 ± 2.0 μg/mL and 6.0 ± 1.8 μg/mL, respectively, in cow milk and 3.4 ± 1.4 μg/mL and 3.4 ± 1.3 μg/mL, respectively, in goat milk). The contents of FAs C16:0, C18:1 (n-9), C12:0 and C14:0 in cow milk measured in the present work are in agreement with previously reported data. La Nasa et al. [12] studied five FFAs in cow milk using a HPLC-ESI-Q-ToF approach after derivatization and the values reported were 40 μg/g, 16 μg/g, 2.5 μg/g and 6.4 μg/g for FAs C16:0, C18:1 (n-9), C12:0 and C14:0, respectively. A disagreement was observed for the content of C18:1 (n-9), for which La Nasa et al. [12] reported a very low value of 0.042 μg/g. Our results for cow milk are also in general agreement with those reported by Amer et al. [10], employing an in-solution derivatization approach followed by GC/MS analysis, where eleven fatty acids were studied and C16:0, C18:1 and C18:0 (n-9) were found to be the major components, followed by C14:0 and C12:0. No data were found in literature for FFA content in goat milk.

**Figure 3:**
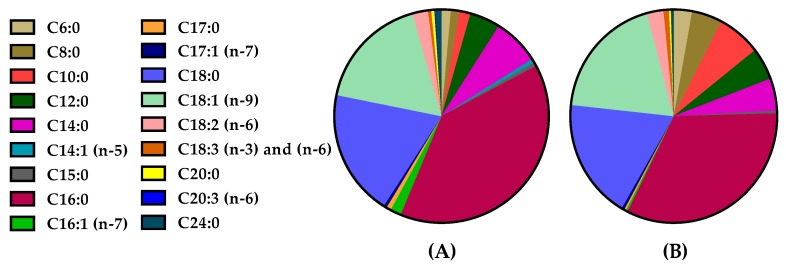
Free fatty acid average content in cow milk (**A**) and goat milk (**B**).

Other fatty acids, either saturated or unsaturated, were found in lower quantities. The relative average contents of FFAs are illustrated in Figure 3A (cow milk) and Figure 3B (goat milk). Comparing goat milk with cow milk, we observed that the content of C18:1 (n-9) is lower in goat milk than in cow milk, while the short chain saturated FAs C6:0, C8:0 and C10:0 were found in higher quantities in goat milk than in cow milk. In contrast, the long chain saturated FAs C20:0 and C24:0, not previously included in any study, are present in higher quantities in cow milk than in goat milk.

The contents of FAs C18:2 (n-6) and total C18:3 (n-3) and (n-6) are similar in cow milk and goat milk (2.0 ± 1.2 μg/mL and 0.4 ± 0.3 μg/mL in cow milk and 1.8 ± 0.6 μg/mL and 0.6 ± 0.2 μg/mL in goat milk, respectively). Polyunsaturated FAs, such as C20:3 (n-6), C20:4 (n-6), C20:5 (n-3), C22:5 (n-3), C22:6 (n-6), were essentially absent from both cow and goat milk samples.

As discussed in the introduction, it is becoming clear that each particular FA may play a different role in human health [6,16,17,18]. In the case of saturated FAs, odd-chain saturated C15:0 and C17:0 have shown to be associated with a lower risk of type 2 diabetes [18]. The levels of odd-chain FFAs C15:0 and C17:0 were estimated and found to be 0.5 ± 0.2 μg/mL and 0.6 ± 0.1 μg/mL in cow milk, respectively and 0.3 ± 0.0 μg/mL and 0.2 ± 0.0 μg/mL in goat milk, respectively. Furthermore, the not-widely abundant monounsaturated FAs C14:1 (n-5), C16:1 (n-7) and C17:1 (n-7) were found at mean values of 0.5 ± 0.4 μg/mL, 1.4 ± 0.7 μg/mL and 0.4 ± 0.0 μg/mL, respectively in cow milk and at lower values (varying from lower than LOQ to 0.3 ± 0.0 μg/mL) in goat milk.

The present method has the advantage of avoiding any derivatization and is the first LC-HRMS method used for this application, which provides a rapid and simultaneous determination of twenty-two FFAs in milk. These are clear advantages over the previously reported methods, which require a derivatization step before analysis and have been applied for the study of a limited number of FAs in milk. The method developed in this work may also find applications for the determination of FFAs in various food sources, after the appropriate validation in each food matrix. 

## 3. Materials and Methods

### 3.1. Chemicals and Reagents

All solvents used were of LC-MS analytical grade. Acetonitrile was purchased from Carlo Erba (Val De Reuil, France), isopropanol and methanol from Fisher Scientific (Laughborough, UK) and formic acid 98–100% from Chem-Lab (Zedelgem, Belgium). Caproic acid was purchased from Alfa Aesar (> 98%,Lancashire, UK), caprylic acid from Sigma Aldrich (>99.5%, Steinheim, Germany), capric acid from Sigma Aldrich (>99%, Steinheim, Germany), lauric acid from Acros Organics (>99%, Geel, Belgium), myristic acid from Sigma Aldrich (>99.5%, Steinheim, Germany), myristoleic acid from Sigma Aldrich (>99%, Steinheim, Germany), pentadecanoic acid from Sigma Aldrich (>99%, Steinheim, Germany), palmitic acid from Fluka (analytical standard, Karlsruhe, Germany), 9-palmitoleic acid from Fluka (analytical standard, Karlsruhe, Germany), margaric acid from Sigma Aldrich (>98%, Steinheim, Germany), 10-Z-heptadecenoic acid from Cayman Chemical Company (>98%, Ann Arbor, MI, USA), stearic acid from Fluka (analytical standard, Karlsruhe, Germany), oleic acid from Fluka (analytical standard, Karlsruhe, Germany), linoleic acid from Sigma Aldrich (>99%, Steinheim, Germany), linolenic acid from Sigma Aldrich (>99%,Steinheim, Germany), arachidic acid from Cayman Chemical Company (>98%, Ann Arbor, MI, USA), bihomo-γ-linolenic acid from Cayman Chemical Company (>98%, Ann Arbor, MI, USA), arachidonic acid from Sigma Aldrich (>99, Steinheim, Germany), 5,8,11,14,17-Z-eicosapentanoic acid from Fluka (analytical standard, Karlsruhe, Germany), 7,10,13,16,19-cis-docosapentaenoic acid from Cayman Chemical Company (>98%, Ann Arbor, MI, USA), 4,7,10,13,16,19-cis-docosahexaenoic acid from Sigma Aldrich (>98%, Steinheim, Germany) and lignoceric acid from Cayman Chemical Company (>98%, Ann Arbor, MI, USA).

### 3.2. Stock and Working Solutions

Stock solutions of the standard compounds were prepared at a concentration of 1000 mg/L in methanol and stored at 4 °C. Working standard solutions (500 ng/mL) were prepared daily by appropriate dilution.

### 3.3. Instrumentation

LC-MS/MS measurements were performed with an ABSciex Triple TOF 4600 (ABSciex, Darmstadt, Germany) combined with a micro-LC Eksigent (Eksigent, Darmstadt, Germany) and an autosampler set at 5 °C and a thermostated column compartment. Electrospray ionization (ESI) in negative mode was used for all the MS experiments. The data acquisition method consisted of a TOF-MS full scan *m/z* 50–850 and several information dependent acquisition (IDA)-TOF-MS/MS product ion scans using 40 V collision energy (CE) with 15 V collision energy spread (CES) used for each candidate ion in each data acquisition cycle (1091). This workflow allows quantitation (primarily using TOF-MS) and confirmation (using IDA-TOF-MS/MS) in a single run. Halo C18 2.7 μm, 90 Å, 0.5 × 50 mm^2^ from Eksigent was used as a column and the mobile phase consisted of a gradient (A: acetonitrile/0.01% formic acid/isopropanol 80/20 *v*/*v*; B: H_2_O/0.01% formic acid). The elution gradient adopted started with 5% of phase B for 0.5 min, gradually increasing to 98% in the next 7.5 min. These conditions were kept constant for 0.5 min, and then the initial conditions (95% solvent B, 5% solvent A) were restored within 0.1 min to re-equilibrate the column for 1.5 min for the next injection (flow rate: 55 µL/min). The data acquisition was carried out with MultiQuant 3.0.2 and PeakView 2.1 from AB SCIEX (ABSciex, Darmstadt, Germany).

EICs were obtained with the use of MultiQuant 3.0.2, which creates the base peak chromatograms for the masses that achieve a mass accuracy window of 5 ppm. The relative tolerance of the retention time was set within a margin of ±5% for caproic and caprylic acid and a margin of ±2.5% for all the rest fatty acids.

### 3.4. Sample Preparation

One milliliter of milk was weighed in a screw cap glass centrifuge tube and methanol (4 mL) was added. The sample was stirred for about 30 s and then centrifuged at 4000× *g* for 10 min. The supernatant (500 μL) was then mixed with 500 μL of water in a vial and this mixture was used for the LC-MS/MS analysis AB SCIEX (ABSciex, Darmstadt, Germany).

### 3.5. Method Validation

The linearity and the limits of detection (LOD) and quantification (LOQ) were assessed. Solutions from 10–500 ng/mL of caproic acid, caprylic acid, capric acid, arachidic acid, bihomo-γ-linolenic acid, arachidonic acid, 5,8,11,14,17-*Z*-eicosapentanoic acid, 7,10,13,16,19-*cis*-docosapentaenoic acid, 4,7,10,13,16,19-cis-docosahexaenoic acid and lignoceric acid (3 replicates; 9 levels (10, 30, 50, 80, 100, 200, 300, 400, 500 ng/mL); *n* = 3 × 9), solutions from 10–700 ng/mL of lauric acid, myristic acid, myristoleic acid, pentadecanoic acid, 9-palmitoleic acid, margaric acid, 10-*Z*-heptadecenoic acid, linoleic acid and linolenic acid (3 replicates; 11 levels (10, 30, 50, 80, 100, 200, 300, 400, 500, 600, 700 ng/mL); *n* = 3 × 11), solutions from 10–1300 ng/mL of palmitic acid, stearic acid and oleic acid (3 replicates; 14 levels (10, 50, 80, 100, 200, 300, 400, 500, 600, 700, 800, 900, 1000, 1300 ng/mL); *n* = 3 × 14). LOD and LOQ were calculated using the signal-to-noise method. A signal-to-noise ratio (S/N) of three is generally accepted for estimating LOD and signal-to-noise ratio of ten is used for estimating LOQ. This method is commonly applied to analytical methods that exhibit baseline noise.

For the recovery and intra-day and inter-day precision, milk samples were spiked at three different concentration levels.

### 3.6. Milk Samples

Twenty brand products of fresh whole milk were collected from the local market in Athens, Greece. Thirteen of them were cow milk products and seven of them goat milk products.

## 4. Conclusions

In conclusion, we present herein the first LC/HRMS method for the determination of FFAs in milk. Our method involves mild sample preparation conditions, excluding the hydrolysis of esterified fatty acids of triacylglycerols or other lipid classes and avoids time-consuming extraction pre-separation, or derivatization procedures. It is rapid and robust, permitting the quantification of twenty-two FFAs in a 10-min single run. The method was applied to thirteen cow milk samples and seven goat milk samples. Τhe long chain saturated FAs C16:0 and C18:0, as well as mono unsaturated C18:1 (n-9), were found as the most abundant FFAs in both cow and goat milk samples. FAs with shorter chains, including C6:0, C8:0 and C10:0, were found in higher quantities in goat milk than in cow milk. The levels of odd-chain FFAs C15:0 and C17:0, which have been shown very recently to be important for human health by lowering the risk of type 2 diabetes, were estimated in cow and goat milk. The method developed in this work may also be used for other applications involving the determination of FFAs in various food sources, after the appropriate validation in each food matrix. 

## Figures and Tables

**Table 1:** Fatty acid standards used in the chromatographic method and mass spectral data.

Compound	Structure	Theoretical Mass [M − H]^−^	Measured Mass [M − H]^−^	Mass Error (ppm)
Caproic acid	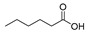	115.0765	115.0764	0.87
Caprylic acid	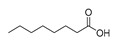	143.1078	143.1075	2.10
Capric acid	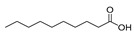	171.1391	171.1390	0.58
Lauric acid	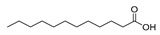	199.1704	199.1703	0.50
Myristoleic acid	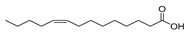	225.1850	225.1849	0.44
Myristic acid	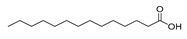	227.2017	227.2016	0.44
Pentadecanoic acid	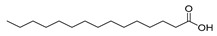	241.2173	241.2171	0.83
Palmitic acid	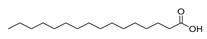	255.2330	255.2326	1.57
9-Palmitoleic acid	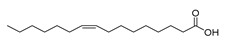	253.2173	253.2168	1.97
Margaric acid	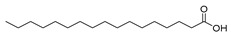	269.2486	269.2485	0.37
10-Z-Heptedecenoic acid	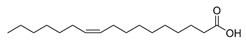	267.2330	267.2331	0.37
Stearic acid	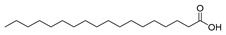	283.2643	283.2641	0.71
Oleic acid	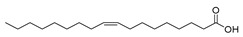	281.2486	281.2485	0.36
Linoleic acid	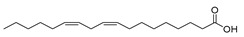	279.2330	279.2330	0.00
Linolenic acid	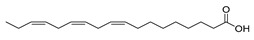	277.2173	277.2175	0.72
Arachidic acid	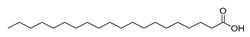	311.2956	311.2955	0.32
Bishomo-γ-linolenic acid	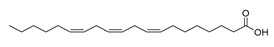	305.2486	305.2487	0.33
Arachidonic acid	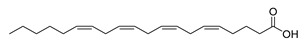	303.2330	303.2330	0.00
5,8,11,14,17-Z-Eicosapentaenoic acid	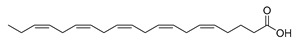	301.2173	301.2174	0.33
7,10,13,16,19-Docosapentaenoic acid	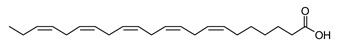	329.2486	329.2485	0.30
4,7,10,13,16,19-Docosahexaenoic acid	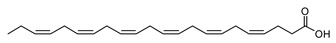	327.2330	327.2327	0.92
Lignoceric acid	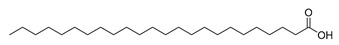	367.3582	367.3583	0.27

**Table 2:** Calibration curve data as well as limits of detection (LOD) and quantification (LOQ).

Analyte	Range (ng/mL)	Calibration Εquations	Linearity (R^2^)	LOD (ng/mL)	LOQ (ng/mL)
Caproic acid	10–500	y = 2 x + 10	0.997	0.5	1.5
Caprylic acid	10–500	y = 34 x − 1773	0.99	0.5	1.1
Capric acid	10–500	y = 93 x − 3009	0.991	0.5	1.7
Lauric acid	10–700	y = 63 x + 2375	0.999	0.6	1.8
Myristic acid	10–700	y = 101 x + 2396	0.998	0.6	1.8
Myristoleic acid	10–700	y = 107 x − 683	0.999	0.6	1.8
Pentadecanoic acid	10–700	y = 78 x + 1319	0.998	0.8	2.4
Palmitic acid	10–1300	y = 55 x + 93651	0.993	0.9	2.3
9-Palmitoleic acid	10–700	y = 194 x + 716	0.999	1.6	4.8
Margaric acid	10–700	y = 93 x + 1668	0.997	0.8	2.4
10-*Z*-Heptedecenoic acid	10–700	y = 192 x − 922	0.999	0.8	2.4
Stearic acid	10–1300	y = 65 x + 98565	0.993	0.9	2.8
Oleic acid	10–1300	y = 167 x + 8786	0.997	0.7	2.3
Linoleic acid	10–700	y = 349 x − 2572	0.998	0.6	1.8
Linolenic acid	10–700	y = 282 x − 2587	0.99	0.6	1.8
Arachidic acid	10–500	y = 77 x − 461	0.994	0.8	2.4
bishomo-γ-Linolenic acid	10–500	y = 627 x − 3622	0.999	0.6	1.8
Arachidonic acid	10–500	y = 855 x − 2450	0.996	0.6	1.8
Eicosapentanoic acid	10–500	y = 825 x − 7182	0.998	0.6	1.8
7,10,13,16,19-Docosapentaenoic acid	10–500	y = 71 x − 238	0.996	0.4	1.2
4,7,10,13,16,19-Docosahexaenoic acid	10–500	y = 563 x − 6428	0.996	0.4	1.2
Lignoceric acid	10–500	y = 141x + 6107	0.996	0.6	1.8

**Table 3:** Accuracy (recovery %) and precision data (RSD %) in spiked milk samples.

Analyte	t_R_ (min)	Spike Level (ng/mL)	Recovery (%)	RSD_r_ (%)	RSD_R_ (%)
Caproic acid	2.2	50 300 500	84 85 93	5.82 2.23 6.56	6.55 3.15 7.89
Caprylic acid	3.4	50 300 500	83 83 93	2.61 0.52 15.47	4.21 1.26 16.68
Capric acid	4.3	50 300 500	82 82 94	6.25 1.00 14.31	8.21 2.36 15.21
Lauric acid	5.0	50 300 700	93 95 96	5.50 5.95 0.47	6.12 6.33 1.54
Myristic acid	5.8	50 300 700	97 98 101	5.97 7.96 12.40	6.12 8.31 13.01
Myristoleic acid	5.2	50 300 700	90 91 91	1.25 0.33 3.07	1.98 1.35 3.81
Pentadecanoic acid	6.1	50 300 700	94 95 95	3.10 3.41 5.41	2.93 3.50 6.02
Palmitic acid	6.4	50 300 700	94 95 95	1.00 0.49 1.50	1.33 1.15 2.34
9-Palmitoleic acid	5.9	50 300 700	90 90 92	1.21 0.31 3.50	1.65 1.63 4.21
Margaric acid	6.6	50 300 700	94 95 101	0.85 0.80 3.69	1.11 1.23 4.81
10-Z-Heptedecenoic acid	6.2	50 300 700	90 90 93	4.31 5.33 3.69	5.12 6.48 4.12
Stearic acid	6.9	50 300 700	94 95 102	3.41 10.08 6.12	4.71 12.47 6.52
Oleic acid	6.5	50 300 700	95 95 95	2.50 2.72 5.80	3.89 4.57 6.33
Linoleic acid	6.1	50 300 700	90 90 94	3.74 4.06 4.51	4.99 5.24 5.18
Linolenic acid	5.7	50 300 700	94 94 94	4.55 4.37 3.43	5.12 6.41 5.78
Arachidic acid	7.3	50 300 500	97 98 100	7.52 8.07 15.64	8.33 9.12 16.01
Bishomo-γ-linolenic acid	6.2	50 300 500	97 97 98	6.21 5.36 15.67	7.46 6.91 16.01
Arachidonic acid	6.0	50 300 700	92 93 96	4.51 4.19 3.97	7.01 6.31 5.96
Eicosapentanoic acid	5.6	50 300 500	91 92 94	6.21 3.10 18.51	8.74 3.41 19.12
7,10,13,16,19- Docosapentaenoic acid	6.1	50 300 500	90 90 93	5.33 4.98 14.55	6.87 5.01 17.81
4,7,10,13,16,19- Docosahexaenoic acid	5.9	50 300 500	93 94 96	2.55 0.41 11.26	5.02 2.51 12.26
Lignoceric acid	8.0	50 300 500	94 95 101	3.21 2.23 6.56	6.87 2.45 7.81

t_R_: retention time; RSDr: intra-day relative standard deviation; RSD_R_: inter-day relative standard deviation.

**Table 4:** Contents of free fatty acids in cow milk and goat milk samples (μg/mL fresh milk).

	Cow Milk (*n* = 13)			Goat Milk (*n* = 7)		
Fatty Acid	Minimum Value (μg/mL)	Maximum Value (μg/mL)	Mean Value ± SD (μg/mL)	Minimum Value (μg/mL)	Maximum Value (μg/mL)	Mean Value ± SD (μg/mL)
C6:0	0.5	2.3	1.2 ± 0.4	1.4	2.8	2.0 ± 0.5
C8:0	0.7	1.9	1.1 ± 0.3	2.2	4.9	3.3 ± 0.9
C10:0	0.6	2.8	1.4 ± 0.6	3.0	5.8	4.7± 1.5
C12:0	1.6	8.8	3.9 ± 2.0	1.8	5.3	3.4 ± 1.4
C14:0	3.5	10.5	6.0 ± 1.8	1.2	5.2	3.4 ± 1.3
C14:1 (n-5)	0.1	1.6	0.5 ± 0.4	<LOQ	<LOQ	<LOQ
C15:0	0.2	0.8	0.5 ± 0.2	0.2	0.3	0.3 ± 0.0
C16:0	26.7	55.1	33.1 ± 8.2	20.6	24.1	22.8 ± 1.8
C16:1 (n-7)	0.5	2.9	1.4 ± 0.7	0.02	0.4	0.2 ± 0.1
C17:0	0.5	0.9	0.6 ± 0.1	0.1	0.2	0.2 ± 0.0
C17:1 (n-7)	0.3	0.4	0.4 ± 0.0	0.3	0.3	0.3 ± 0.0
C18:0	7.1	26.0	16.5 ± 5.3	8.6	16.9	12.7 ± 2.8
C18:1 (n-9)	9.3	19.8	14.8 ± 3.8	13.1	13.4	13.3 ± 0.3
C18:2 (n-6)	0.1	4.1	2.0 ± 1.2	1.2	2.4	1.8 ± 0.6
C18:3 (n-3) and (n-6)	0.1	0.9	0.4 ± 0.3	0.3	0.8	0.6 ± 0.2
C20:0	0.3	0.6	0.4 ± 0.1	0.2	0.3	0.2 ± 0.0
C20:3 (n-6)	0.1	0.1	0.1 ± 0.0	<LOQ	<LOQ	<LOQ
C20:4 (n-6)	<LOD	<LOD	<LOD	<LOD	<LOD	<LOD
C20:5 (n-3)	<LOQ	<LOQ	<LOQ	<LOQ	<LOQ	<LOQ
C22:5 (n-3)	<LOD	<LOD	<LOD	<LOD	<LOD	<LOD
C22:6 (n-3)	<LOD	<LOD	<LOD	<LOD	<LOD	<LOD
C24:0	0.6	0.9	0.8 ± 0.1	0.3	0.4	0.3 ± 0.0

<LOQ: lower of limit of quantification; <LOD: lower of limit of detection; SD: standard deviation.

## References

[1] Jensen R.G. (2002). The composition of bovine milk lipids: January 1995 to December 2000. J. Dairy Sci..

[2] German J.B., Dillard C.J. (2006). Composition, structure and absorption of milk lipids: A source of energy, fat-soluble nutrients and bioactive molecules. Crit. Rev. Food Sci. Nutr..

[3] Deeth H.C., Fitz-Gerald C.H., Fox P.F. (1995). Lipolytic enzymes and hydrolytic rancidity in milk and dairy products. Advanced Dairy Chemistry.

[4] Antonelli M.L., Curini R., Scricciolo D., Vinci G. (2002). Determination of free fatty acids and lipase activity in milk: Quality and storage markers. Talanta.

[5] Deeth H.C. (2006). Lipoprotein lipase and lipolysis in milk. Int. Dairy J..

[6] Gómez-Cortés P., Juárez M., Angel de la Fuente M. (2018). Milk fatty acids and potential health benefits: An updated vision. Trends Food Sci. Technol..

[7] Mannion D.T., Furey A., Kilcawley K.N. (2016). Free fatty acids quantification in dairy products. Int. J. Dairy Technol..

[8] Christie W.W. (1998). Gas chromatography–mass spectrometry methods for structural analysis of fatty acids. Lipids.

[9] Liua Z., Ezernieksa V., Rochforta S., Cocks B. (2018). Comparison of methylation methods for fatty acid analysis of milk fat. Food Chem..

[10] Amer B., Nebel C., Bertram H.C., Mortensen G., Hermansen K., Dalsgaar T.K. (2013). Novel method for quantification of individual free fatty acids in milk using an in-solution derivatisation approach and gas chromatography-mass spectrometry. Int. Dairy J..

[11] Mannion D.T., Furey A., Kilcawley K.N. (2019). Development and validation of a novel free fatty acid butyl ester gas chromatography method for the determination of free fatty acids in dairy products. J. Agric. Food Chem..

[12] La Nasa J., Degano I., Brandolini L., Modugno F., Bonaduce I. (2018). A novel HPLC-ESI-Q-ToF approach for the determination of fatty acids and acylglycerols in food samples. Anal. Chim. Acta.

[13] Zhou T., Leng J., Peng Y., Zhang L., Guo Y. (2016). Mass spectrometric analysis of free fatty acids in infant milk powders by frozen pretreatment coupled with isotope-labeling derivatization. J. Sep. Sci..

[14] Simeoni M.C., Sergi M., Pepe A., Mattocci E., Martino G., Compagnone D. (2018). Determination of free fatty acids in cheese by means of matrix solid-phase dispersion followed by ultra-high performance liquid chromatography and tandem mass spectrometry analysis. Food Anal. Methods.

[15] World Health Organization Draft Guidelines on Saturated Fatty Acid and Trans-Fatty Acid Intake for Adults and Children. Public Consultation May to June 2018. https://extranet.who.int/dataform/upload/surveys/183439/files/Public-Consultation-on-the-Draft-Global-Strategy-on-Digital-Health.pdf.

[16] Astrup A., Bertram H.C.S., Bonjour J.-P., de Groot L.C.P., de Oliveira Otto M.C., Feeney E.L., Garg M.L., Givens I., Kok F.J., Krauss R.M. (2019). WHO draft guidelines on dietary saturated and trans fatty acids: Time for a new approach?. BMJ.

[17] De Oliveira Otto M.C., Lemaitre R.N., Song X., King I.B., Siscovick D.S., Mozaffarian D. (2018). Serial measures of circulating biomarkers of dairy fat and total and cause-specific mortality in older adults: The cardiovascular health study. Am. J. Clin. Nutr..

[18] Imamura F., Fretts A., Marklund M., Ardisson Korat A.V., Yang W.-S., Lankinen M., Qureshi W., Helmer C., Chen T.-A., Wong K. (2018). Fatty acid biomarkers of dairy fat consumption and incidence of type 2 diabetes: A pooled analysis of prospective cohort studies. PLoS Med..

